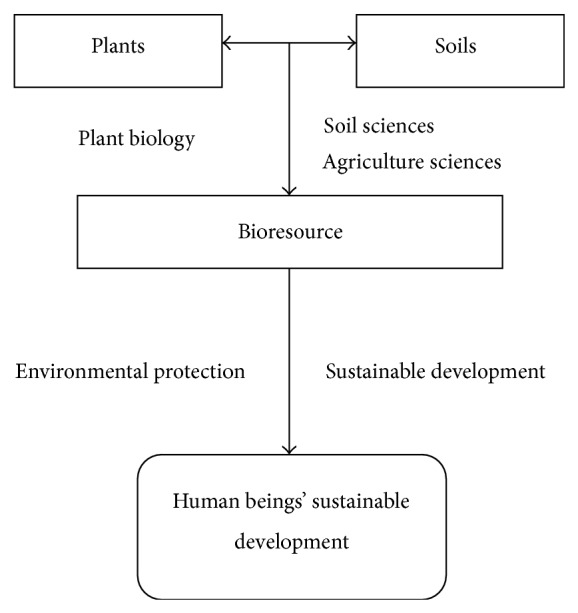# Plant Abio-Stress and Bioresources Utilization for Sustainable Development

**DOI:** 10.1155/2014/163123

**Published:** 2014-12-31

**Authors:** Hong-bo Shao, Marian Brestic, Si-Xue Chen, Zhao Chang-Xing, Xu Gang

**Affiliations:** ^1^Institute of Biotechnology, Jiangsu Academy of Agricultural Sciences, Nanjing 210014, China; ^2^Yantai Institute of Coastal Zone Research (YIC), Chinese Academy of Sciences (CAS), Yantai 264003, China; ^3^Department of Plant Physiology, Slovak Agricultural University, Tr. A. Hlinku 2, 949 01 Nitra, Slovakia; ^4^Cancer and Genetics Research Complex, University of Florida, 2033 Mowry Road, Room 438, Gainesville, FL 32610, USA; ^5^College of Agronomy and Plant Protection, Qingdao Agricultural University, Qingdao 266109, China

Resources, environment, food, and sustainable development (REFS) are the topics of the world. There are many factors in the current world that limit plant productivity and resources utilization including objective and subjective aspects. More efforts should be made to know the physiological mechanisms for plants responding to abiotic stresses such as salt, heat, drought, cold, and UV-B, which have been extensively investigated under increasing global climate change. How to efficiently use bioresources and protect and construct eco-environment for sustainable development is the greatest challenge. As known, plants can provide human beings with renewable energy, food, and materials and are the base for sustainable development in different forms around the world. The other related issues are bioresources efficient utilization and eco-environmental construction. So, these major global challenges are the precondition of our sustainable survival.

Plants have evolved different mechanisms for adapting themselves to different stress during long-term natural evolution and domestic pressure, which at least include molecular, biochemical, physiological, cellular, organ, tissue, anatomy, individual, and ecological scales. The physiological level is very important as it is the key for farmers to fertilize and manage crops. More recent progress related with molecular biology and metabolism and bioresources and eco-environment has also taken place for the past 20 years. How to regulate plant-soils relationship for sustainable plant productivity at precise level has a long way to go not only in agriculture but also in environmental sciences. In the topic issue, many papers are devoted to this field such as soil, microbiological organisms, green-house gas, vegetation restoration, and salt soil improvement with more potential measures. Soil resources utilization is another important aspect. In soil P bioavailability is a key issue. In this special issue, some papers have been devoted to this issue and its role in eco-restoration. Partial papers about salt-resistant genes characterization and functional analysis will be of great value to further gene resources utilization in agriculture and salt soil improvement. We hope that readers of this special issue will find not only accurate data and updated articles that are involved in REFS, but also important questions to be resolved, for example, by different biomeasures. Related relationship can be referenced to Figure 1.

*Hong-bo Shao* *Hong-bo Shao* *Marian Brestic* *Marian Brestic* *Si-Xue Chen* *Si-Xue Chen* *Zhao Chang-Xing* *Zhao Chang-Xing* *Xu Gang* *Xu Gang*

## Figures and Tables

**Figure 1:**